# Supplementary material for: The translatome of quiescent Plasmodium falciparum gametocytes reveals parasite pyridoxal 5’-phosphate (PLP) biosynthesis is essential for efficient mosquito stage development
Source: PLoS Pathog. 2026 Sep 23;22(9):e1014635. doi: 10.1371/journal.ppat.1014635 (PMC13626478; doi:10.1371/journal.ppat.1014635)
Supplement: Supplementary file 1 [file ppat.1014635.s007.pdf]

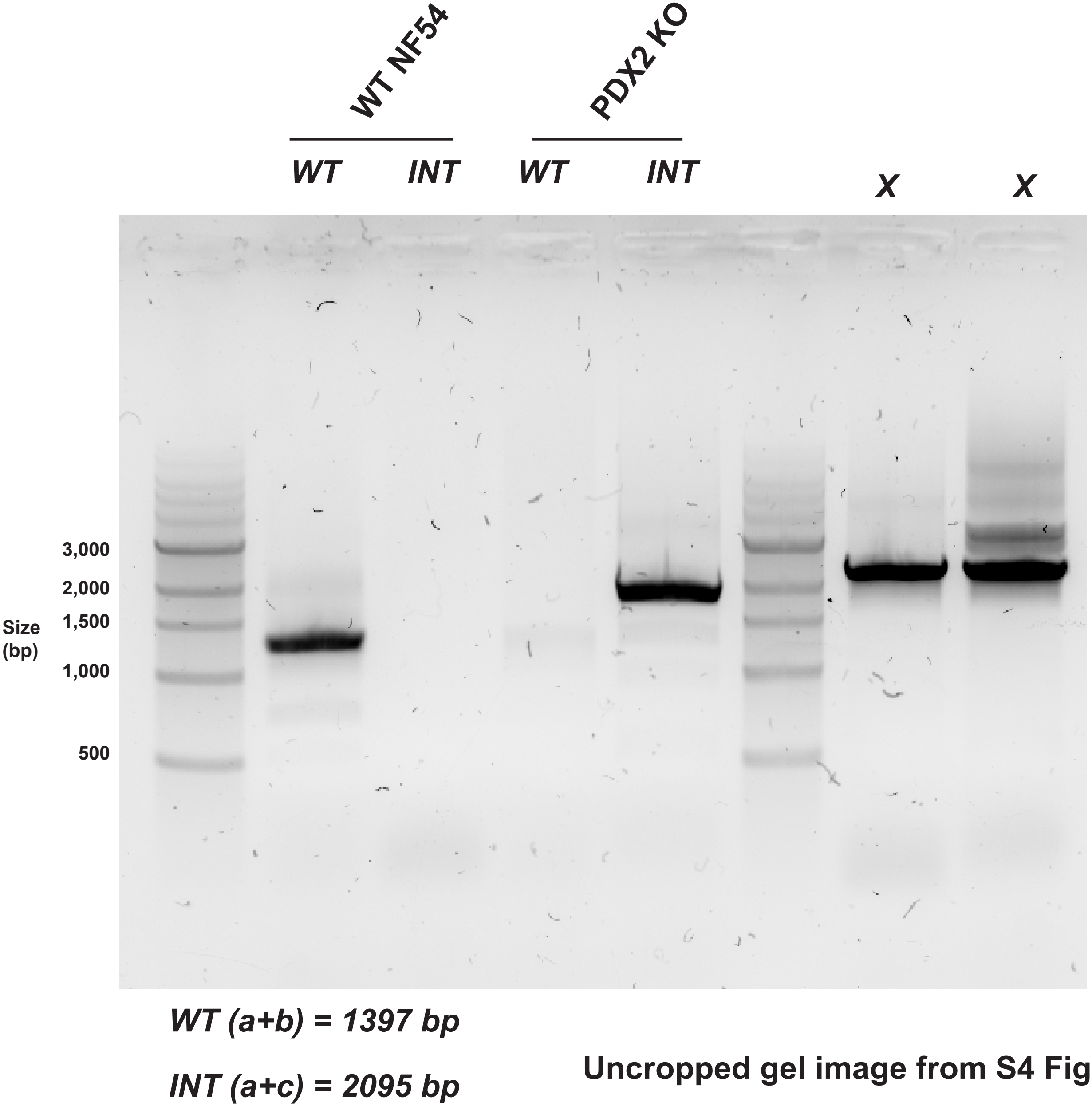

Uncropped gel image from S4 Fig
