# Supplementary material for: The translatome of quiescent Plasmodium falciparum gametocytes reveals parasite pyridoxal 5’-phosphate (PLP) biosynthesis is essential for efficient mosquito stage development
Source: PLoS Pathog. 2026 Sep 23;22(9):e1014635. doi: 10.1371/journal.ppat.1014635 (PMC13626478; doi:10.1371/journal.ppat.1014635)
Supplement: Supplementary file 2 [file ppat.1014635.s020.docx]

| Sequence  5’-3’ | Description |
| --- | --- |
| catattaagtatataatattatagggg  tattatcactacaaggtagagctagaa  atagcaagt | Forward primer for gRNA integration by Gibson cloning. |
| acttgctatttctagctctaccttgtagt  gataatacccctataatattatatactt  aatatg | Reverse primer for gRNA integration by Gibson cloning. |
| tgcggttcaaaatcacgttgtta  tccgtcgactttatacaattcatc | Forward primer to amplify mNeonGreen and complement PDX2 repair sequence. |
| aactataggggtattatcactaatg  gcaagtttgccagcaac | Reverse primer to amplify mNeonGreen and complement the PDX2 5’ homology region for Gibson cloning. Also used to confirm genome integration (S4 Fig. - Primer c). |
| acaacgtgattttgaaccgcatataa  atcattttatcaaattacaaatacc | Forward primer to amplify the PDX2 repair sequence, introduce a point mutation to eliminate the PAM site for the gRNA. |
| tagctatgtcgactatccgcgtacttc  atctgataatatttctcttatataagg | Reverse primer to amplify the PDX2 repair sequence and complement the CRISPR plasmid for Gibson cloning. |
| agtgataatacccctatagttatttctgac | Reverse primer to amplify the 5’ homology region. |
| atagcaaaagaaaagaaagcggta  ttatcataatatccagaagcac | Forward primer to amplify the 5’ homology region and complement the CRISPR plasmid for Gibson cloning. Also used to confirm assess either WT genotype or PDX2KO integration (S4 Fig. - Primer a) |
| gttcaaaatcaccttgtagtgataatac | Primer targeting only the native WT *pdx2* sequence. Used to confirm WT genotype (S4 Fig. - Primer b) |
